# Application of Pulsed Ohmic Heating for Rapid Cooking of Indica Rice: Quality Enhancement and Mechanistic Insights

**DOI:** 10.3390/foods15152742

**Published:** 2026-08-04

**Authors:** Haiqian Xu, Guifeng Wei, Boru Chen, Jian Li, Lang-Hong Wang, Xin-An Zeng, Sudhir K. Sastry

**Affiliations:** 1 Guangdong Provincial Key Laboratory of Intelligent Food Manufacturing, School of Food Science and Engineering, Foshan University, Foshan 528225, China; 15819320870@163.com (H.X.); 15278834036@163.com (G.W.); brchen@fosu.edu.cn (B.C.); li_jian@fosu.edu.cn (J.L.); 2 Department of Food, Agricultural and Biological Engineering, The Ohio State University, 590 Woody Hayes Drive, Columbus, OH 43210, USA; sastry.2@osu.edu

**Keywords:** ohmic heating, rice, gelatinization, microstructure, moisture distribution

## Abstract

Rice cooking is a time-consuming process, and conventional heating methods often suffer from low heating efficiency and non-uniform heat transfer, which may compromise cooking quality. Although pulsed ohmic heating (OH) has shown potential for accelerating food heating, its effects on the quality formation and structural evolution of cooked rice remain insufficiently understood. Therefore, this study applied OH technology to improve the cooking efficiency and quality of indica rice. Through single-factor and response surface methods, the optimal OH parameters were determined: 18 min treatment time, 9.4 μs pulse width, 0.52 kV/cm electric field strength, and 265 Hz frequency. Under these conditions, the cooking time was significantly reduced by 28% compared to traditional rice cookers, achieving a comprehensive quality score of 88.42 points. Optimized OH treatment improved water absorption and volume expansion ratio, reduced hardness, and enhanced the overall flavor characteristics, accompanied by changes in the microstructure and starch organization. Multi-scale structural analyses indicated that OH treatment was associated with accelerated starch gelatinization, reduced starch crystallinity, enhanced moisture migration, and possible alterations in starch–protein interactions, which were consistent with the observed improvements in rice texture. Importantly, at the 18 min maturation point, the estimated glycemic index of OH-treated rice showed no significant difference compared with traditionally cooked rice (83.69 vs. 82.88, *p* > 0.05), ensuring nutritional quality. These findings suggest that OH is a promising technology for improving rice cooking efficiency and quality while maintaining nutritional properties.

## 1. Introduction

As one of the world’s most important staple crops, rice serves not only as a core staple in the Chinese diet, but its processing and cooking quality directly impacts the nutritional intake and quality of life for hundreds of millions of people. Indica rice, a significant variety within this category, is widely favored for its high amylose content, slender grain shape, and tendency to separate during cooking. However, in traditional steaming processes, heat transfer primarily relies on external sources conducting and convecting heat through water to the grain interior. This “surface-to-core” energy transfer often results in overheating of the grain surface while the core remains under-hydrated, leading to uneven cooking, a stiff texture, and the loss of heat-sensitive nutrients [1]. Furthermore, although modern electric rice cookers have improved in thermal efficiency (typically ranging from 78% to 88% for mainstream models), the energy transfer during the cooking process still primarily relies on external sources conducting and convecting heat through water to the grain interior. This ‘surface-to-core’ heat transfer mode often leads to energy losses and uneven cooking. In today’s society pursuing sustainable development and energy conservation, the development of novel, highly efficient rice processing technologies has become particularly urgent [2].

Pulsed ohmic heating (OH), also known as Joule heating, is a volumetric heating technology that directly converts alternating current into thermal energy by leveraging the electrical resistance properties of food materials. In the present study, a pulsed current mode with adjustable pulse width and pulse frequency was employed while maintaining Joule heating as the dominant heating mechanism. Currently, this technology is widely applied in food sterilization [3], extraction [4], modification [5], drying [6], fermentation [7], and other fields. In recent years, numerous scholars have employed OH technology for rice cooking, finding no significant difference in textural properties compared to traditional methods. Previous studies have suggested that OH has the potential to improve heating efficiency compared with conventional thermal processes under appropriate operating conditions; however, its actual energy performance is strongly influenced by equipment design, electrical characteristics of food materials, and processing parameters [8,9,10].

Although previous studies have demonstrated that OH can effectively accelerate rice cooking and influence starch gelatinization, moisture migration, texture, and microstructural characteristics, most investigations have primarily focused on individual quality attributes or processing performance. A comprehensive understanding of how rapid volumetric heating affects water redistribution, multi-scale starch structural evolution, possible changes in starch–protein spatial organization, and their relationships with cooking quality remains limited. Therefore, this study combined response surface optimization with multi-scale structural characterization to systematically investigate the relationships among rapid volumetric heating, water migration, starch structural evolution, microstructural changes, rheological behavior, and cooking quality during OH processing of indica rice. The findings provide further insights into the quality formation of OH-cooked rice and offer scientific support for the development of rapid and high-quality rice cooking technologies.

## 2. Materials and Methods

### 2.1. Materials

Guangdong Simiao rice (indica rice, IR), a representative long-grain indica rice cultivar, was purchased from Guangzhou Lingnan Suiliang Grain Co., Ltd. (Guangzhou, Guangdong Province, China). The rice was produced in Guangzhou, harvested in 2026, and complied with the Chinese National Standard for Rice (GB/T 1354) [11]. The samples were commercially polished rice and were vacuum-packaged and stored under cool and dry conditions for 7 days before OH treatment. The rice grains exhibited typical slender characteristics of indica rice, with an approximate grain length of 6.5–7.5 mm, width of 2.0–2.3 mm, and a length-to-width ratio of approximately 3.0–3.5. The proximate composition of IR was as follows: moisture, 13.50%; protein, 7.18%; fat, 1.93%; total starch, 79.59%; and amylose, 23.03%.

Total starch and amylose content assay kits were purchased from Beijing Solarbio Technology Co., Ltd. All other analytical-grade chemical reagents were purchased from legitimate commercial suppliers. Deionized water was used throughout the experiments.

### 2.2. OH Treatment and Sample Preparation

**Figure 1:**
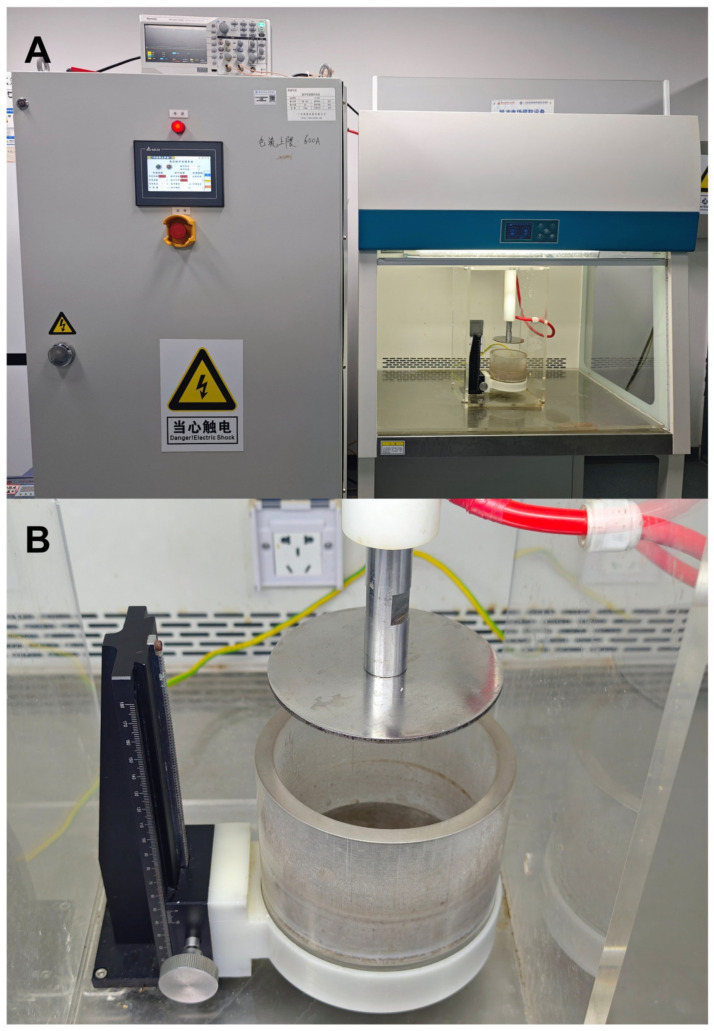
Schematic of the experimental device employed for the OH treatment. Overview of the OH system, showing the control cabinet (left) and the transparent treatment chamber (right) (**A**). Close-up view of the electrode assembly and treatment chamber (**B**).

A custom-built pulsed ohmic heating (OH) system (Guangzhou Paihu Technology Co., Ltd., Guangzhou, China) was used (Figure 1). The system was equipped with an adjustable charging voltage (0–800 V), pulse frequency (0–300 Hz), and pulse width (0–10 μs). The charging voltage refers to the input voltage of the pulse-generation module, whereas the instantaneous voltage applied to the treatment chamber during pulse discharge was determined by the preset electric field strength. The OH treatment chamber consisted of a cylindrical vessel (10 cm diameter × 10 cm height) equipped with two parallel titanium electrodes. The electrode spacing was fixed at 3 cm, and the effective working volume of the chamber was approximately 785 mL. The electric field strength was calculated according to the following equation:

$$E=\frac{V}{d}$$

where E represents the electric field strength (kV/cm), V represents the actual applied voltage between electrodes (kV), and d represents the electrode spacing (cm).

The applied voltage, current, and corresponding electrical parameters under different electric field strengths are summarized in Table S1. During OH processing, electrical energy was delivered in a pulsed mode. The pulse width and pulse frequency were adjusted to regulate the energy delivery profile. The voltage waveform generated by the pulsed power supply exhibited a rectangular pulse pattern, characterized by a rapid voltage rise, a short constant-voltage plateau, and subsequent voltage decay. The temperature increase during treatment was primarily generated through Joule heating caused by the electrical resistance of the rice–water system.

The estimated electrical energy input during pulsed OH treatment was calculated based on the applied voltage, maximum current, pulse width, pulse frequency, and treatment duration according to the following equation:

$$Q=V\times I_{max}\times PW\times f\times t$$

where Q represents the estimated electrical energy input (J), V represents the applied voltage (V), $I_{max}$ represents the maximum current (A), PW represents the pulse width (s), f represents the pulse frequency (Hz), and t represents the treatment duration (s).

For each experiment, 170 g of indica rice was placed into the OH treatment chamber, followed by the addition of 150 mL of deionized water. The rice–water mixture was directly cooked within the OH system under the preset electric field strength, pulse width, pulse frequency, and treatment time. No additional heating or conventional cooking step was applied after OH treatment. After completion of OH treatment, excess water was removed, and the cooked rice samples were immediately collected for subsequent analyses.

Untreated raw rice without OH treatment or conventional cooking was used as the untreated control group (CK). Rice prepared using the “Quick Cook” mode of a commercially available rice cooker (Model MB-WFS4037, Midea, Foshan, China) (170 g rice plus 150 mL water, steamed for 25 min) was used as the conventional cooking control group (CK-25).

After OH treatment, a portion was immediately used as a fresh sample for subsequent analysis (e.g., texture, color, sensory evaluation, etc.); another portion was drained of moisture and placed in a vacuum freeze dryer (VFD-2000A, Shanghai Xinnuo Instrument Group Co., Ltd., Shanghai, China) for freeze-drying at −50 °C for 48 h, to be used for subsequent analysis. The freeze-dried samples were ground into rice flour using a multifunctional grinder (FW-135, Tianjin Teste Instrument Co., Ltd., Tianjin, China), sieved through a 100-mesh screen, and stored in sealed containers for subsequent analysis.

### 2.3. Experimental Design

#### 2.3.1. Effects of OH Treatment on Rice Cooking

To investigate the fundamental effects of OH treatment, the processing parameters were selected based on preliminary experiments considering heating stability, rice integrity, and cooking performance. The OH conditions were fixed at an electric field strength of 0.30 kV/cm, pulse frequency of 300 Hz, and pulse width of 10 μs. Treatment times were set at 10, 12, 14, 16, 18, and 20 min, and the corresponding samples were designated as IROH-10 to IROH-20, respectively. Water absorption, swelling ratio, texture, color, gelatinization state, and in vitro digestibility were measured and compared with CK-25 to clarify the influence of treatment time on the cooking of indica rice. The results obtained in this stage were further used as the basis for subsequent process optimization.

#### 2.3.2. Optimization of the OH Conditioning Process for Rice

Based on the finding that OH treatment significantly promotes the ripening of indica rice, the OH process parameters were systematically optimized to further achieve optimal eating quality and ripening results. First, single-factor experiments were conducted to determine the optimal ranges for each key parameter: treatment duration, pulse width, electric field strength, and pulse frequency on the gelatinization degree and sensory scores of indica rice. The design of the single-factor experiments is summarized in Table 1. Based on the results of the single-factor experiments, four key parameters—treatment duration (A), pulse width (B), electric field strength (C), and pulse frequency (D)—were selected. Gelatinization degree and sensory score were selected as complementary indicators representing starch structural transformation and consumer-oriented eating quality, respectively. Since both parameters are critical for evaluating rice cooking performance, an equal weighting strategy was adopted to balance physicochemical maturation and sensory acceptability. A similar comprehensive evaluation strategy integrating gelatinization degree and sensory evaluation with equal weighting has also been reported for optimizing food processing conditions [12]. Therefore, a composite score calculated from gelatinization degree and sensory score, each accounting for 50% of the total weight, was used as the response variable for the Box–Behnken response surface design. The factor levels are shown in Table S2. The optimal process combination was determined through 29 experimental runs and subsequently validated.

### 2.4. Evaluation of the Quality and Processing of Ohm-Thermal-Cured Indica Rice

#### 2.4.1. Qualitative Assessment of Gelatinization Status and Quantitative Determination of Gelatinization Degree

The gelatinization characteristics of indica rice were evaluated both qualitatively and quantitatively. The gelatinization status, representing the macroscopic completion of cooking, was determined using the pressing method according to GB/T 25226-2010 [13]. The degree of gelatinization, reflecting the proportion of starch converted from an ordered granular structure to a gelatinized state, was determined using a modified enzymatic hydrolysis method based on the procedure reported by Liu [14].

Briefly, 0.2 g of rice powder was accurately weighed and transferred into a 100 mL conical flask, followed by the addition of 25 mL distilled water. The suspension was heated in a boiling water bath for 30 min to ensure complete gelatinization and then cooled to room temperature. Subsequently, 2 mL of 3% (*w*/*v*) α-amylase solution was added, and enzymatic hydrolysis was performed at 39 °C for 90 min. After hydrolysis, 2 mL of 1 mol/L HCl and 2 mL of 1 mol/L NaOH were sequentially added for reaction termination and pH adjustment. The volume was then adjusted to 50 mL with distilled water. After centrifugation at 6000 rpm for 15 min, the supernatant was collected for reducing sugar determination. For DNS colorimetric analysis, 1.0 mL of the supernatant was transferred into a 25 mL volumetric tube, followed by the addition of 1.0 mL distilled water and 1.5 mL DNS reagent. The mixture was heated in boiling water for 5 min and then diluted to 25 mL with distilled water. The absorbance was measured at 540 nm. The degree of gelatinization was calculated according to Equation (1), based on the relative reducing sugar release between treated rice samples and raw rice flour.

(1) $$Degree\;of\;Gelatinization\;(\%)=\frac{A1}{A2}\times 100$$

where A1 is the absorbance of rice after OH treatment, A2 is the absorbance of raw rice that has not undergone OH treatment.

#### 2.4.2. Determination of Water Absorption and Volume Expansion

The method described by Boluda-Aguilar [15] was followed with minor modifications. The weight and volume of rice samples were measured before and after treatment. The water absorption ratio (WAR) and volume expansion ratio (VER) were calculated based on the changes in weight and volume, respectively. Water absorption and volume expansion were calculated as follows:

(2) $$WAR(\%)=\frac{\left(W1-W0\right)}{W0}\times 100$$

(3) $$VER(\%)=\frac{\left(V1-V0\right)}{V0}\times 100$$

where W0 is the weight of the rice (mg), W1 is the weight of the treated rice (mg), V0 is the volume of the 5 g rice sample (mL), and V1 is the volume of the treated rice sample (mL).

#### 2.4.3. Color Measurement

The color characteristics of cooked rice samples were measured using a portable colorimeter (CR-10, Konica Minolta, Tokyo, Japan). Before measurement, the instrument was calibrated using the standard white calibration plate provided by the manufacturer. Color parameters were determined based on the CIE Lab* color system under a standard illuminant D65 with a 2° standard observer angle. The measurements were performed using a diffuse illumination geometry (8°/d) with a circular measurement aperture (8 mm diameter). Freshly cooked rice samples were evenly placed in a transparent sample container, and color values were directly measured on the surface of the samples. The whiteness index (*W*) was calculated according to the following equation:

(4) $$W=100\sqrt{(100-L^{*}{)}^{2}+a^{*2}+{b^{*}}^{2}}$$

where L* represents the lightness of the sample surface, a* represents the redness or greenness of the sample surface, b* represents the yellowness or blueness of the sample surface, and *W* represents the whiteness of the rice.

#### 2.4.4. Texture Properties

The method described by Liu [16] was followed with minor modifications. Ten samples of processed fresh rice were placed on the texture analyzer platform and tested using the TPA mode. Texturometer parameter settings: a P/36R cylindrical probe was selected, with pre-test, test, and post-test speeds of 2.0 mm/s, 1.0 mm/s, and 2.0 mm/s, respectively; a deformation ratio of 70%; and a probe contact force of 5.0 g. Each sample was tested five times in parallel.

#### 2.4.5. Determination of Reducing Sugars

The reducing sugar content was determined according to the method described by Zeng [17] with slight modifications. An amount of 1.0 g of cooked rice sample was mixed with 3 mL distilled water (solid-to-liquid ratio of 1:3, g/mL) in a 15 mL centrifuge tube. The mixture was extracted at 50 °C with continuous shaking for 20 min to facilitate the release of reducing sugars. After extraction, the suspension was centrifuged at 8000 rpm for 10 min, and the supernatant was collected for analysis. The reducing sugar content was determined using the 3,5-dinitrosalicylic acid (DNS) colorimetric method. Briefly, 0.5 mL of supernatant was mixed with 2 mL DNS reagent and heated in a boiling water bath for 5 min. After cooling to room temperature, the mixture was diluted to 10 mL with distilled water, and the absorbance was measured at 540 nm. Glucose was used as the calibration standard, and the reducing sugar content was calculated based on the glucose standard curve. All measurements were performed in five independent experiments, and results were expressed as mean ± standard deviation. The standard used here was glucose (y = 1.372x + 0.005, R^2^ = 0.9991). The reducing sugar contents were calculated using the following formula:

(5) $$Reducing\;sugar(mg/g)=\frac{\left(C\times V\right)}{m}$$

where C represents the concentration of reducing sugars (mg/mL) calculated from the standard curve, V represents the total volume of the extract (mL), and m represents the mass of the sample (g).

#### 2.4.6. Determination of Starch Components

The in vitro starch digestibility, expressed as the contents of rapidly digestible starch (RDS), slowly digestible starch (SDS), and resistant starch (RS), was determined following the method of Zeng [17]. The freeze-dried flour samples underwent enzymatic hydrolysis with α-amylase and glucoamylase. The glucose released at specific time intervals (0, 20, and 120 min) was quantified, and the RDS, SDS, and RS fractions were calculated based on the hydrolysis kinetics.

#### 2.4.7. Determination of Starch Hydrolysis Rate

Following the method described in Section 2.4.6, samples were taken at 0, 20, 60, 90, and 120 min into the digestion to measure glucose release, and the starch hydrolysis rate was calculated.

#### 2.4.8. Determination of the Estimated Glycemic Index

Based on the results of the starch hydrolysis rate determination in Section 2.4.7, a curve was plotted showing the relationship between time and starch hydrolysis rate. The area under this curve represents the extent to which the sample affects blood glucose levels after digestion. Using the hydrolysis rate of white bread as the reference (set at 100%), the hydrolysis index (HI) and the estimated glycemic index (eGI) were calculated as follows:

(6) $$HI(\%)=\frac{AUC\;sample}{AUC\;white\;bread}\times 100$$

(7) eGI=0.549HI+39.71

where AUC is the area under the hydrolysis curve.

#### 2.4.9. Sensory Evaluation

The sensory evaluation of cooked rice was conducted according to GB/T 15682-2008 [18]. A panel consisting of 10 trained food science students with previous experience in sensory evaluation of rice products was recruited for the evaluation. Before assessment, all cooked rice samples were randomly coded with three-digit numbers, and the panelists were blinded to the specific treatment information. The samples were presented in a randomized order to minimize the influence of tasting sequence. The sensory attributes, including aroma, appearance, texture, palatability, and overall acceptability, were evaluated using a standardized scoring system with a maximum score of 100 points. The detailed sensory evaluation criteria and scoring scheme are provided in Table S3. Each sample was evaluated independently by all panelists, and the average score was calculated for statistical analysis.

#### 2.4.10. Electronic Tongue and Electronic Nose Analysis

For these tests, we used the method described by Huang [19] with minor modifications. An amount of 20 g of prepared indica rice was placed in a beaker; 200 mL of distilled water was added to each beaker, allowed to soak for 30 min, then centrifuged and filtered. Then, 100 mL of the filtrate was transferred for electronic tongue analysis.

We adapted the method described by Park [20] with minor modifications. An amount of 5 g of prepared indica rice was placed in a 25 mL headspace vial and equilibrated at 70 °C for 30 min. E-nose analysis parameters: carrier gas was dry air; flow rate was set to 150 mL/min, temperature to 80 °C, acquisition time to 120 s, and retention time to 600 s.

### 2.5. Investigation of the Structural Evolution During OH Treatment of Indica Rice

To gain a deeper understanding of the underlying mechanisms, all analyses in this section were conducted using the optimal OH parameter combination obtained through response surface optimization (pulse width 9.4 μs, electric field strength 0.52 kV/cm, pulse frequency 265 Hz). To dynamically observe the maturation process, a treatment time gradient of 10, 12, 14, 16, 18, and 20 min was established; the corresponding samples were designated as OH-10 to OH-20, and changes in their moisture content, microstructure, and starch structure were systematically analyzed. Rice prepared using the “Quick Cook” mode of a commercially available rice cooker, as described earlier, was used as the traditional cooking control group (CK-25).

#### 2.5.1. Temperature Profile Analysis During OH Treatment

To evaluate the heating behavior of the OH system, the temperature profiles of indica rice during OH treatment and conventional rice cooker cooking were monitored under their respective processing conditions. For OH treatment, temperature measurements were conducted under the optimized parameters obtained from response surface optimization. The temperature at the geometric center of the rice–water system was recorded at 20 s intervals throughout the treatment process using a digital temperature probe. For comparison, the temperature profile of rice cooked using the “Quick Cook” mode of a commercial rice cooker was also recorded under the same rice-to-water ratio conditions.

#### 2.5.2. Low-Field Nuclear Magnetic Resonance (LF-NMR) and Magnetic Resonance Imaging (MRI) Analysis

Following the method reported by Srikaeo [21], a low-field nuclear magnetic resonance analyzer (Model NMI20-060H-I, Suzhou Xinmai Analytical Instrument Co., Ltd., Suzhou, China) was used to determine the water-binding state of rice samples. A 5.00 g sample of processed fresh rice was sealed in an NMR tube, and the transverse relaxation time (T2) was measured using the Carr–Purcell–Meiboom–Gill (CPMG) pulse sequence. Specific parameter settings: 90° (P1) and 180° (P2) pulse widths were 6.00 μs and 22.56 μs, respectively; the wait time (TW) was 3000 ms; and the number of scans (NS) was 4.

Following the method described by Wang [22], processed whole rice grain samples were placed in 10 mm NMR tubes and subjected to proton density-weighted imaging using a low-field NMR spectrometer (NMI20-060H-I, Suzhou Xinmai Analytical Instrument Co., Ltd., Suzhou, China). The imaging parameters were set as follows: repetition time (TR) 1000 ms, echo time (TE) 6.96 ms, 256 cumulative scans, receive gain (RG) 20 dB, preamplifier gain (PRG) 3, and digital receive gain (DRG) 5. The acquired images were processed using Niumag NMR software (V3.0) and converted into false-color images for analysis.

#### 2.5.3. Scanning Electron Microscopy (SEM)

Following the method described by Wang [23], freeze-dried rice grain samples were cut and mounted on the sample stage using conductive adhesive. After ion sputtering gold coating, the samples were examined using a scanning electron microscope (Phenom XL, Thermo Scientific, Waltham, MA, USA) to observe their microstructure. The cross-sections and surface structures of the samples were observed at magnifications of 200× and 1000×, respectively.

#### 2.5.4. Thermal Properties

The procedure was adapted from Kang [24] with minor adjustments. A 4 mg sample of freeze-dried powder was weighed and placed in an alumina crucible within a thermogravimetric analyzer. The parameters were set as follows: nitrogen flow rate of 20 mL/min, heating rate of 10 °C/min, and temperature range of 30–600 °C. The first-derivative curve of the thermogravimetric curve was calculated.

#### 2.5.5. Fourier Transform Infrared Spectroscopy (FTIR)

Following the method described by Li [25], a Fourier transform infrared spectrometer (Nicolet iN10: Thermo Scientific, Waltham, MA, USA) was used to analyze the freeze-dried powder samples. The wavelength range was set to 400–4000 cm^−1^, with 32 scans and a resolution of 4 cm^−1^. OMNIC software (Version 8.2, Thermo Fisher Scientific, Waltham, MA, USA) was used to analyze the short-range ordered structure of the rice samples in the 800–1200 cm^−1^ wavelength range.

#### 2.5.6. X-Ray Diffraction (XRD)

Following the method described by Han [26] with minor modifications, XRD analysis was performed on the freeze-dried powder samples. Test parameters: diffraction angle (2θ) scan range 10–40°; scan step 0.08°. The relative crystallinity of the samples was calculated using MID Jade software (Version 6.0, Materials Data, Livermore, CA, USA).

#### 2.5.7. Rheological Properties

The rheological properties of cooked rice samples were determined according to the method described by Villanueva [27] with slight modifications. The cooked rice samples obtained after different treatments were directly used for rheological measurements. Before testing, the samples were carefully transferred onto the rheometer platform and allowed to equilibrate for 5 min to ensure temperature stabilization and structural relaxation. Rheological measurements were performed using a multi-functional rheometer (MCR 702e, Anton Paar (Shanghai) Trading Co., Ltd., Shanghai, China) equipped with a cone-and-plate measuring system. A cone with a diameter of 40 mm and a cone angle of 1° was used, and the gap distance was set at 1.8 mm. A preliminary strain sweep test was performed to determine the linear viscoelastic region (LVR), and a strain amplitude of 0.2% was selected for subsequent frequency sweep measurements. Frequency sweep tests were conducted at 25 °C over a frequency range of 0.1–10 Hz. The storage modulus (G′), loss modulus (G″), and loss tangent (tanδ = G″/G′) were recorded to characterize the viscoelastic properties of cooked rice samples.

#### 2.5.8. Changes in the Morphology and Distribution of Rice Starch and Proteins

Following the method described by Zhu [28] with modifications, a confocal laser scanning microscope (CLSM, LSM800, Zeiss, Oberkochen, Germany) was used to observe the distribution of starch and proteins in the samples. The treated rice grains were frozen in liquid nitrogen, embedded, and sectioned to a thickness of 8 μm. The sections were stained using a 1:1 (*v*/*v*) ethanol mixture containing 0.4% fluorescein isothiocyanate (FITC) and 0.04% rhodamine B (RhB). The stained sections were mounted on slides and observed under a CLSM using a 10× objective. The acquired images were analyzed using ImageJ software (version 1.54, National Institutes of Health, Bethesda, MD, USA).

### 2.6. Statistical Analysis

Statistical analysis was performed using SPSS Version 17.0 software (SPSS Inc., Chicago, IL, USA). All experiments were conducted with five independent replicates, and each replicate represented an independent rice cooking process. For each replicate, 10 rice grains were randomly selected from the cooked samples for measurement. Data are presented as mean ± standard deviation (SD). One-way analysis of variance (ANOVA) followed by Duncan’s multiple range test was used to determine significant differences among treatments at a significance level of *p* < 0.05. Prior to ANOVA, the normality of data distribution and homogeneity of variance were evaluated to confirm that the assumptions of ANOVA were satisfied. Response surface methodology (RSM) analysis was performed using Design-Expert 11 software (Stat-Ease Inc., Minneapolis, MN, USA). The adequacy of the quadratic regression model was evaluated based on analysis of variance (ANOVA), including model significance, lack-of-fit test, coefficient of determination (R^2^), adjusted R^2^, and predicted R^2^. Residual analyses, including residual plots and the comparison between predicted and experimental values, were performed to verify model reliability and predictive accuracy.

## 3. Results and Discussion

### 3.1. Preliminary Effects of OH Treatment on the Cooking Characteristics and Quality of Indica Rice

#### 3.1.1. Gelatinization State

**Figure 2:**
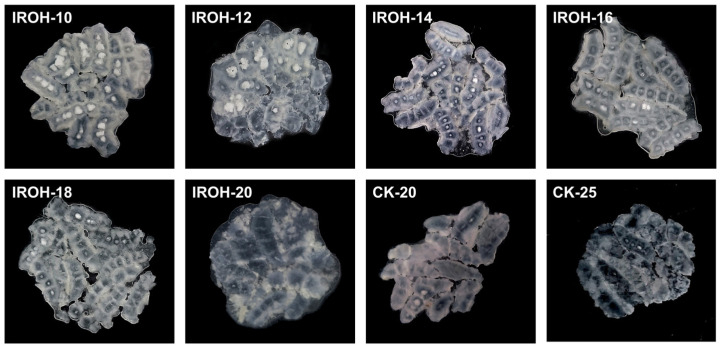
Gelatinization status of indica rice samples after OH treatment. IROH-10 to IROH-20 represent OH treatment for 10–20 min, while CK-20 and CK-25 represent quick cooking for 20 and 25 min, respectively.

Figure 2 shows the gelatinization status of indica rice press-molded samples after different treatments. After 20 min of cooking in a rice cooker (CK-20), some white cores remained in the rice grains, indicating that certain areas had not yet fully gelatinized; however, after 25 min of cooking (CK-25), the rice grains became completely transparent, with no white or opaque spots, indicating that the rice had fully gelatinized. For indica rice subjected to OH, a distinct white core was still visible in the center of the grains initially, indicating that gelatinization had not yet begun. As the OH duration increased, the gelatinization process commenced. When the cooking time was extended to 20 min (IROH-20), the indica rice became completely transparent, and its degree of gelatinization was comparable to that of CK-25. This indicates that short-term OH treatment has a limited effect on the gelatinization of indica rice, but as the treatment time increases, OH significantly promotes the gelatinization of the rice, ultimately bringing it to a fully cooked state. This demonstrates that OH technology can effectively improve the degree of rice cooking, providing a new technical pathway for optimizing rice cooking processes.

#### 3.1.2. Determination of Water Absorption and Volume Expansion Ratio

**Figure 3:**
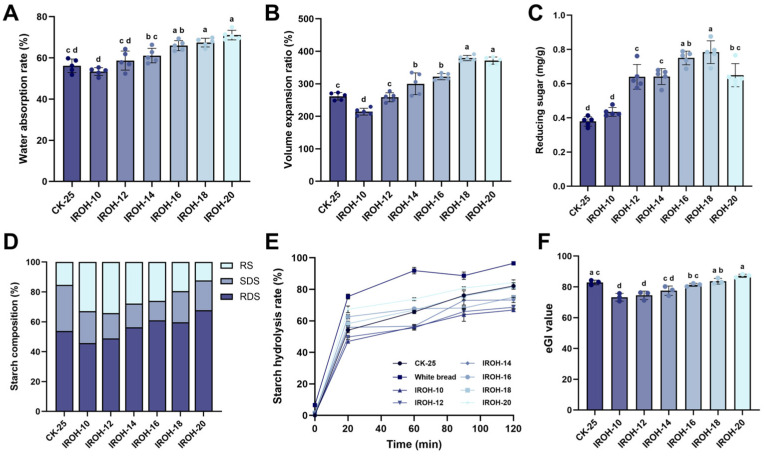
Comprehensive evaluation of OH treatment on the quality attributes of indica rice. Water absorption ratio (**A**), volume expansion ratio (**B**), reducing sugar content (**C**), starch composition (**D**), in vitro starch hydrolysis ratio (**E**), eGI value (**F**). IROH-10 to IROH-20 represent OH treatment for 10–20 min, while CK-20 and CK-25 represent quick cooking for 20 and 25 min, respectively. Different lowercase letters indicate significant differences among treatments (*p* < 0.05).

Water absorption and volume expansion ratio are key indicators of rice’s hydration capacity and structural relaxation. As shown in Figure 3A,B, as the duration of OH treatment increased, the water absorption and volume expansion ratio of indica rice rose from 56.16% and 261.58% to 71.08% and 371.32%. It is worth noting that when the OH treatment time reached 18–20 min, the water absorption ratio was already higher than that of the group steamed for 25 min in a conventional rice cooker (CK-25). This enhancement effect is primarily attributed to two mechanisms. First, OH induces intense molecular thermal motion, thereby increasing the diffusion coefficient of water molecules. This enhanced diffusion likely accelerates the penetration of water into the amorphous regions and crystal defects of starch granules, thereby promoting granule swelling [29]. This interpretation is consistent with the weakening of the ordered starch structure observed by FTIR and XRD, suggesting progressive disruption of double-helical arrangements and crystalline organization during OH treatment. Second, rapid heating generates a slight vapor pressure inside the rice grains, thereby widening the gaps between the originally tightly packed starch granules and providing additional physical pathways for water influx [30].

#### 3.1.3. Colorimetry

The color characteristics of rice samples were evaluated using the L*, a*, b*, and whiteness index (*W*) values. As shown in Table S4, with increasing OH treatment time, the L* and *W* values gradually decreased, whereas the a* value showed an increasing trend, indicating that prolonged OH treatment resulted in a darker appearance and a slight increase in redness of the cooked rice samples. Compared with traditionally cooked rice (CK-25), the OH-treated samples exhibited relatively lower whiteness, particularly after 20 min of treatment, where a slightly grayish-white appearance was observed.

The observed color changes may be related to the combined effects of prolonged heating and moisture redistribution during OH treatment. In addition, thermal reactions such as Maillard reactions between reducing sugars and amino compounds could potentially contribute to color development during rice cooking; however, this hypothesis requires further verification through targeted analyses of Maillard reaction products and related precursors. Previous studies have shown that different heating methods can influence the degree of rice maturation and flavor-related metabolic processes, thereby further affecting its overall color and sensory quality [31].

#### 3.1.4. Textural Properties

Textural properties are a core factor determining the edible quality of rice. Among these, starch—as the primary component of rice—plays a crucial role, with its structure, gelatinization temperature, swelling characteristics, and the exudation of molecules from starch granules serving as key parameters that determine the texture of cooked rice. During cooking, starch granules lose their crystalline structure, absorb water, and swell, while amylose exudes from the granules [32].

Table S5 presents the results of changes in rice textural properties following rice cooker steaming and OHH. As processing time increased, the hardness, chewiness, and stickiness of indica rice all decreased significantly. These observations are consistent with previous reports on OH accelerating the softening of food texture [9,33]. Notably, just 18 min of OH reduced the hardness of indica rice to approximately 343.86 gf, significantly lower than the 508.84 gf observed after 25 min of rice cooker treatment. This rapid softening phenomenon can be attributed not only to the accelerated expansion and gelatinization of starch granules induced by volumetric OH, but also to the loosening of the cell wall–middle lamella matrix surrounding rice endosperm cells. Rice endosperm cells are surrounded by a cell wall structure rich in non-starch polysaccharides and matrix components [34]. During OH treatment, enhanced water diffusion and rapid internal heating may weaken the tight organization between adjacent endosperm cells, thereby promoting structural loosening and accelerating texture softening. Furthermore, the springiness and cohesiveness of the samples decreased after ohmic treatment, likely due to rapid volumetric heat generation promoting starch swelling and molecular rearrangement, thereby modifying intermolecular interactions within the gel network [35]. However, this structural relaxation actually enhances the ready-to-eat palatability of the cooked rice, giving it a soft and sticky texture that better aligns with modern consumer preferences [8].

#### 3.1.5. Analysis of Reducing Sugar Content

The increase in reducing sugar content during the OH treatment process is closely related to the increased gelatinization and structural disruption of starch granules under rapid volumetric heating conditions. The disruption of the ordered starch structure increases the likelihood of thermodegradation and hydrolysis of starch chains, thereby promoting the formation of short-chain fragments and reducing ends [36]. As shown in Figure 3C, the reducing sugar content initially increased and then decreased with increasing treatment time. At the beginning of the treatment (10 min), the reducing sugar content of indica rice was relatively low (0.43 mg/g). Subsequently, the reducing sugar content gradually increased and reached a maximum value of 0.78 mg/g at 18 min, followed by a slight decrease with prolonged treatment. The reducing sugar content of OH-treated rice remained higher than that of the control group throughout the treatment process. This phenomenon may be related to the enhanced hydration and structural loosening of starch granules induced by OH treatment, which facilitated the exposure of reducing groups within the starch matrix. The subsequent decrease in reducing sugar content after 20 min may be associated with further thermal reactions and structural rearrangement during prolonged OH treatment. However, the specific chemical pathways responsible for this reduction require further investigation.

#### 3.1.6. In Vitro Digestion Analysis

During enzymatic hydrolysis, starch can be classified into three categories based on its digestion rate and extent: rapidly digestible starch (RDS), slowly digestible starch (SDS), and resistant starch (RS). Among these, RS is not easily broken down by enzymes in the small intestine; SDS is completely but slowly absorbed, helping to maintain stable blood glucose levels; RDS is rapidly digested and absorbed, leading to a rapid rise in blood glucose. As shown in Figure 3D, when the OH treatment time was extended from 10 to 20 min, the proportion of resistant starch in indica rice gradually decreased from 32% to 12%, whereas the proportion of rapidly digestible starch increased significantly to 68%. In contrast, the content of slowly digestible starch first decreased and then increased. This phenomenon may be related to the gradual loosening of the endosperm microstructure induced by prolonged OH treatment. As observed in the scanning electron microscopy images, extended OH treatment promoted the formation of a more porous and loosely structured starch–protein matrix within the rice endosperm. The increased internal porosity and structural disruption likely enhance water penetration and enzyme accessibility to the starch substrate, thereby facilitating hydrolysis and digestion [37].

The nutritional and functional properties of rice, particularly its digestibility, are directly influenced by the degree of starch gelatinization and the content of resistant starch [38]. As shown in the experimental results in Figure 3E, the starch hydrolysis rate increased steadily with increasing OH treatment time. The hydrolysis rate was fastest during the initial 20 min of digestion and then gradually leveled off. This is primarily because OH treatment causes damage to starch granules; the longer the treatment time, the greater the damage, making it easier for water molecules to penetrate the crystalline regions and thereby providing more opportunities for enzymatic reactions [37].

The estimated glycemic index (eGI) is used to assess the extent to which a food affects blood glucose levels in the human body; it reflects the rate at which carbohydrates in food are digested, absorbed, and converted into glucose that enters the bloodstream. As shown in Figure 3F, although OH accelerated the hydrolysis process, at the 18 min treatment point (when the rice was fully matured), its eGI value (83.69) showed no significant difference from that of traditionally steamed indica rice (82.88). This indicates that while this technology improves processing efficiency, it does not adversely affect the glycemic load of the rice, fully demonstrating the mildness and safety of physical modification technology.

### 3.2. Optimization of the Thermal Conditioning Process for Indica Rice and Analysis of Its Quality

#### 3.2.1. Results of Single-Factor Experiments

**Figure 4:**
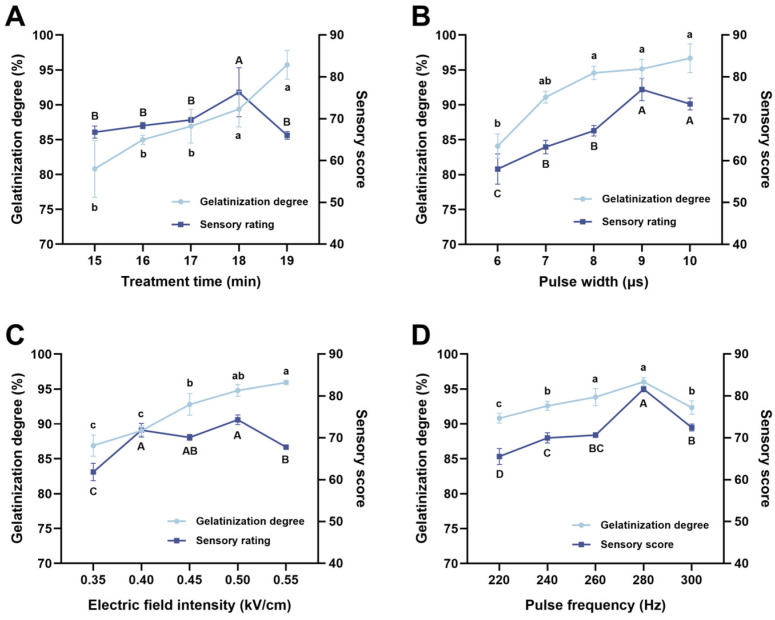
Comprehensive evaluation of OH treatment on the key processing parameters of indica rice. Treatment time (**A**), pulse width (**B**), electric field intensity (**C**), pulse frequency (**D**). Different lowercase or uppercase letters indicate significant differences (*p* < 0.05) between the treatment groups.

To determine the optimal process parameters using the response surface method, single-factor experiments were conducted to measure the following four influencing factors: pulse width, treatment duration, electric field strength, and pulse frequency. As shown in Figure 4A, as the treatment duration increased from 15 min to 19 min, the gelatinization degree of indica rice continued to rise, reaching a peak of 97.95% at 19 min. However, at this point, the rice grains exhibited severe structural breakdown due to excessive water absorption, and the sensory score dropped significantly from the optimal value of 74 points at 18 min. This indicates that excessive heat accumulation leads to a deterioration in textural quality.

Figure 4B illustrates the effect of pulse width on the degree of gelatinization and sensory evaluation. An increase in pulse width significantly increases the energy input per pulse. However, when the pulse width exceeded 9 μs, the sensory evaluation score began to decline. This may be attributed to suboptimal heating dynamics caused by high energy input: during the early stages of OH, the dry, ungelatinized core of the rice grain exhibits high electrical resistance. A high pulse width causes this region to overheat rapidly, while the inward migration of moisture lags behind, resulting in excessively soft rice and a clumpy texture in the cooled rice, which adversely affects its edible quality.

As shown in Figure 4C, the effect of electric field strength is most pronounced. Within the range of 0.35 to 0.50 kV/cm, the Joule heating effect increases gradually with rising intensity, promoting water penetration into the deeper layers of the endosperm and causing gelatinization to rise rapidly from 88.21% to 95.18%, with a corresponding improvement in sensory performance. However, when the intensity was further increased to 0.55 kV/cm, the excessively high rate of temperature rise triggered uneven volumetric expansion, resulting in damage to the appearance of the rice grains and a decrease in whiteness.

Figure 4D illustrates the effect of pulse frequency on gelatinization degree and sensory scores. As the pulse frequency increased, the gelatinization degree and sensory scores of indica rice first rose and then fell; at 280 Hz, both scores reached their highest values, at 95.07% and 81.67 points, respectively. However, as the pulse frequency continued to increase, both scores decreased. This may be because, during rice processing, an appropriate pulse frequency optimizes the heat generation profile, whereas an excessively high pulse frequency might lead to inefficient energy deposition or altered heat and mass transfer dynamics, thereby limiting the ability of water to fully penetrate the rice grains during cooking. The precise mechanism requires further investigation.

#### 3.2.2. Results of Response Surface Experiments on OH-Treated and Aged Indica Rice

Using Design-Expert 11 software, the experimental data shown in Table 2 were analyzed by response surface methodology. The regression equation for the overall score (Y) was obtained using coded factors, where A, B, C, and D represent coded values of treatment time, pulse width, electric field strength, and pulse frequency, respectively: Y = 89.976 + 1.28917A − 1.06417B + 1.26083C − 0.539167D + 0.0275AB + 0.275AC −0.69AD + 1.3175BC + 0.9825BD − 0.465CD − 3.55133A^2^ − 2.85633B^2^ − 3.19383C^2^ − 3.37633D^2^.

Table 2 shows that the *p*-value for the regression model was less than 0.01, indicating that the quadratic model was highly significant (*p* < 0.01), while the lack-of-fit test was not significant (*p* > 0.05), suggesting that the model adequately fitted the experimental data. The coefficient of determination (R^2^) was 0.9553, with an adjusted R^2^ of 0.9106 and a predicted R^2^ of 0.7879. The difference between adjusted R^2^ and predicted R^2^ was less than 0.2, indicating acceptable predictive consistency of the model. In addition, the coefficient of variation (CV) was 1.07%, and the adequate precision value was 14.854, which was higher than the recommended value of 4, demonstrating an acceptable signal-to-noise ratio. These statistical parameters confirmed the adequacy and reliability of the developed regression model within the experimental design range. Based on the F-values, the relative importance of the factors was ranked as follows: treatment time > electric field strength > pulse width > pulse frequency.

**Figure 5:**
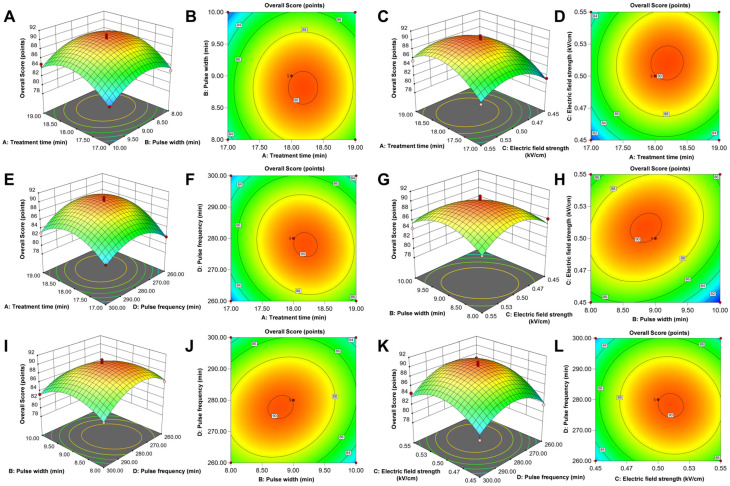
The response surface graph of the influence of various factors on the comprehensive score of indica rice. (**A**) 3D plot of processing time versus pulse width; (**B**) contour plot of processing time versus pulse width; (**C**) 3D plot of processing time versus electric field strength; (**D**) contour plot of processing time versus electric field strength; (**E**) 3D plot of processing time versus pulse frequency; (**F**) contour plot of processing time versus pulse frequency; (**G**) 3D plot of pulse width versus electric field strength; (**H**) Contour plot of pulse width versus electric field strength; (**I**) 3D plot of pulse width versus pulse frequency; (**J**) Contour plot of pulse width versus pulse frequency; (**K**) 3D plot of electric field strength versus pulse frequency; (**L**) Contour plot of electric field strength versus pulse frequency. Each subfigure shows the combined effect of the corresponding factor pair on the comprehensive score, with the optimal region highlighted by a red dot or circle.

As shown in Figure 5, the response surface plot exhibits a trend that first increases and then decreases. The surface for treatment time appears steeper than those for other factors, indicating that treatment time has a significant impact on the comprehensive score of indica rice. Under the influence of interactions, the interactions between pulse width and electric field strength, and between pulse width and pulse frequency, were significant. However, the interactions between treatment time and pulse width, treatment time and electric field strength, treatment time and pulse frequency, and between electric field strength and pulse frequency were not significant. This implies that in actual processing, electrical parameters must be precisely matched to balance energy input with structural integrity.

Through optimization analysis, the optimal process conditions predicted by the response surface model were determined as a treatment duration of 18.413 min, pulse width of 9.417 μs, electric field strength of 0.527 kV/cm, and pulse frequency of 265.242 Hz, with a predicted comprehensive score of 87.721 points. Considering the feasibility of experimental operation, the optimized conditions were adjusted to a treatment time of 18 min, pulse width of 9.4 μs, electric field strength of 0.52 kV/cm, and pulse frequency of 265 Hz for experimental validation. Five independent validation experiments were subsequently conducted under the adjusted optimal conditions. The obtained comprehensive scores were 88.42, 89.13, 88.96, 86.69, and 87.55 points, with an average value of 88.15 ± 1.02 points (*n* = 5). The relative deviation between the experimental mean value and the predicted value was calculated as 0.49%, and the 95% confidence interval of the experimental results was 86.88–89.42 points. The close agreement between the predicted and experimental values confirmed the reliability of the response surface model.

Furthermore, compared with the conventional rice cooker treatment (25 min in quick-cook mode), OH treatment reduced the cooking time of indica rice by approximately 28%. These results demonstrate that OH technology can improve cooking efficiency while maintaining desirable quality characteristics of cooked rice.

#### 3.2.3. Evaluation of Optimal Processing Conditions

##### Cooking Quality and Texture Characteristics

Under the optimal OH treatment, the water absorption and volume expansion ratio of indica rice were significantly higher than those achieved by conventional rice cooker cooking, increasing from 57.28% and 268.25% to 62.72% and 372.54%, respectively (Table S6). Textural analysis further revealed that OH treatment reduced the hardness of indica rice from 504.90 gf to 429.53 gf, while its cohesiveness and springiness also showed a downward trend. This is consistent with the mechanism explained earlier regarding how OH treatment disrupts the starch structure of indica rice.

##### Electronic Tongue and Electronic Nose Analyses

**Figure 6:**
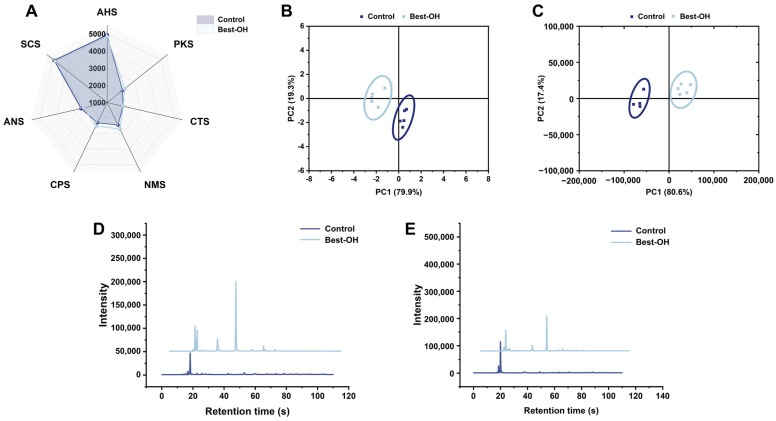
Sensory response analysis of indica rice under the optimal OH treatment conditions. Electronic tongue radar graph and principal component comparison graph (**A**,**B**), electronic tongue principal component comparison graph (**C**), fingerprint spectra C1 and C2 of indica rice (**D**,**E**). AHS, SCS, ANS, PKS, CTS, CPS, and NMS, respectively, represent the sensor response values of the electronic tongue for sourness, bitterness, sweetness, general taste, saltiness, compound taste, and umami. Each data scatter in (**B**,**C**) represents an individual experimental replicate sample. The control group is untreated and quickly cooked for 25 min.

Analysis of the electronic tongue scatter plot (Figure 6A) reveals that OH did not alter the baseline taste profile of indica rice but exhibited distinct advantages in terms of taste intensity. Compared with the control group, OH-treated samples exhibited significantly higher umami and richness response values, suggesting an enhanced taste perception after OH treatment. This improvement may be related to changes in the accessibility and distribution of taste-active components caused by thermal-induced structural modifications during cooking. However, electronic tongue analysis only reflects the overall taste response characteristics and cannot directly identify specific compounds responsible for these changes. Furthermore, principal component analysis (PCA) showed clear separation between the OH-treated group and the control group along the PC1 axis, which explained 79.9% of the total variance (Figure 6B), indicating that OH treatment induced distinct changes in the overall taste characteristics of cooked indica rice.

Electronic nose analysis combined with PCA (Figure 6C) further demonstrated differences in the volatile flavor profiles between OH-treated and conventionally cooked indica rice. The PCA results showed clear discrimination between the two cooking methods, with the rice cooker-treated samples distributed in the third quadrant and OH-treated samples mainly located in the first quadrant. PC1 accounted for 80.6% of the total variance, suggesting that the primary differences in volatile odor fingerprints were associated with the different cooking processes. Furthermore, odor fingerprint analysis (Figure 6D,E) showed distinct response patterns between the two groups during the 30–60 s detection period, indicating that OH treatment modified the overall volatile flavor characteristics of cooked rice. Nevertheless, further chromatographic identification (e.g., GC–MS analysis) would be required to determine the specific volatile compounds responsible for these differences.

### 3.3. Mechanistic Study of Ohmic Thermal Conditioning of Rice

#### 3.3.1. Thermal Evolution During the OH Rice Cooking Process

**Figure 7:**
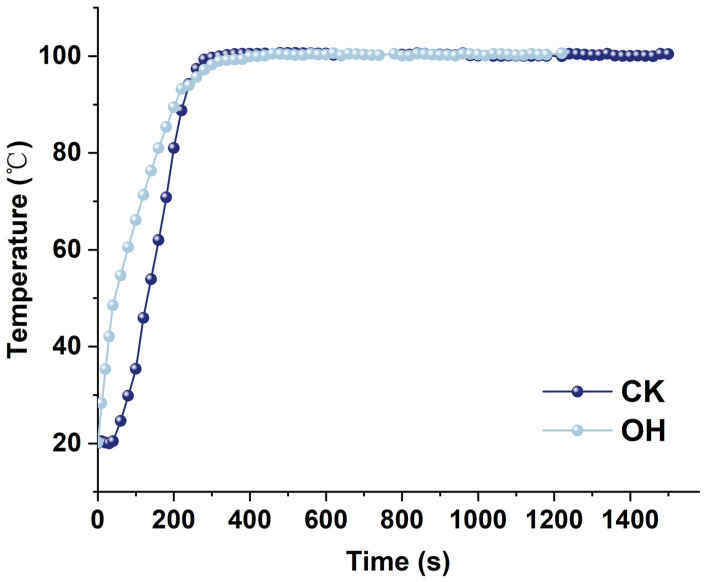
Temperature profiles of indica rice during OH treatment and conventional rice cooker cooking.

As shown in Figure 7, both OH and conventional rice cooker (CK) treatments exhibited a rapid heating stage followed by a stable plateau near 100 °C. However, the OH system showed a significantly faster heating rate during the initial stage. Under the optimized OH conditions, the temperature increased to approximately 90 °C within 200 s, whereas the CK group required a much longer time to reach the same temperature range. This result indicates that OH treatment enabled more rapid and efficient heat transfer throughout the rice–water system.

The accelerated heating behavior is mainly attributed to the volumetric heating mechanism of OH, in which heat is generated directly within the sample matrix through electrical resistance rather than relying solely on external heat conduction and convection. The rapid thermal accumulation likely promoted earlier starch gelatinization, moisture migration, and structural disruption within rice grains, thereby contributing to the improved cooking efficiency. Similar heating characteristics of OH systems have also been reported by Kanjanapongkul [29].

#### 3.3.2. Water Distribution

**Figure 8:**
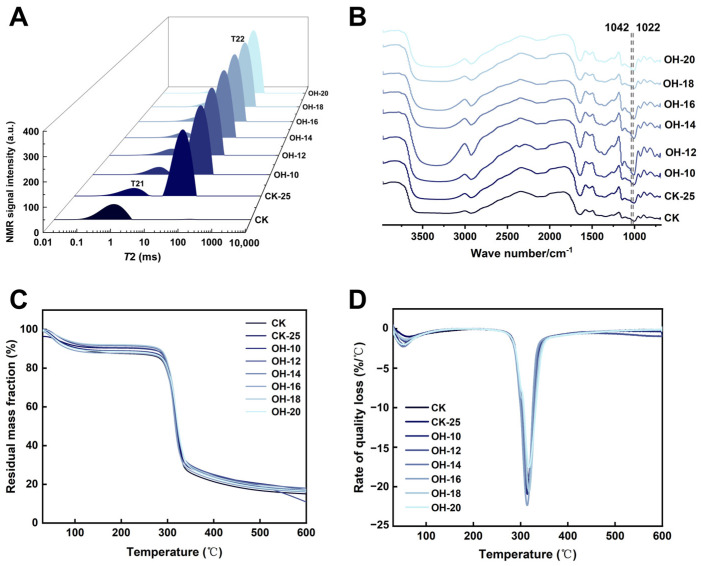
Structural and thermal characterization of indica rice subjected to different processing treatments. Inverse spectrum of longitudinal relaxation time (**A**), infrared spectrum (**B**), thermogravimetric curve (**C**) and differential thermo-gravimetric curve (**D**). The control group is untreated and quickly cooked for 25 min.

LF-NMR can sensitively capture the dynamic behavior of hydrogen protons within a sample, thereby revealing the patterns of water migration. In the T2 relaxation spectrum (Figure 8A), indica rice exhibits two distinct characteristic peaks: T21 (bound water, 0.1–10 ms) and T22 (free water, 10–1000 ms), with corresponding peak areas A21 and A22, representing proton density. As shown in Table S7, with the prolongation of OH treatment time, the total water signal intensity A increased from 7580.24 for OH-10 to 8388.11 for OH-20, representing a 10.66% increase—a significant rise that is far higher than that observed in the conventional cooking group. More importantly, the T21 signal for bound water shifted markedly to the right (from 1.69 ms to 2.38 ms), indicating that the interaction between water molecules and macromolecules such as starch and proteins has weakened, thereby increasing the mobility of water. This phenomenon is primarily attributed to the rapid volumetric heating characteristics of OH treatment. Joule heating is generated directly within the rice grains, thereby accelerating the hydration and gelatinization of the starch. Previous studies have shown that starch gelatinization is accompanied by a weakening of intermolecular hydrogen bonds, the swelling of starch granules, and an increase in water diffusion pathways [29]. Consequently, the increased water molecule mobility observed in this study is consistent with the accelerated hydration process during OH treatment. Furthermore, the presence of a pulsed electric field induces electro-polarization of water molecules. Although this electrical effect is less significant than the Ohmic effect in terms of heat generation, it effectively alters the orientation distribution of water molecules, resulting in a macroscopically accelerated increase in the proportion of free water [39].

#### 3.3.3. Effects of OH on the Moisture Distribution in Indica Rice

**Figure 9:**
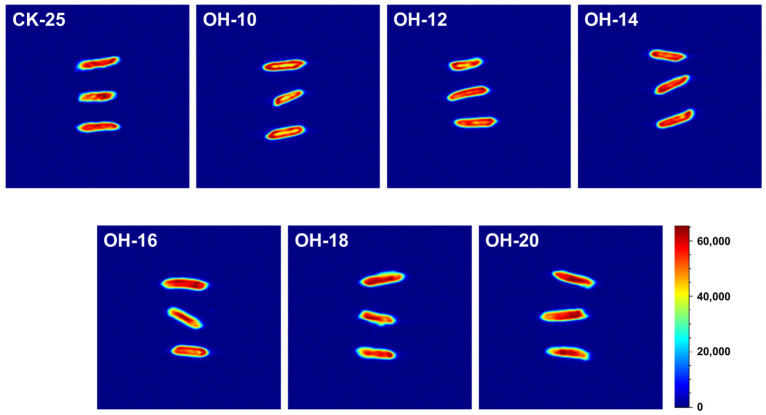
The influence of different treatments on the moisture distribution image of indica rice.

MRI technology further visualized the migration and dynamic distribution of water within the rice grains during the OH treatment process. As clearly shown in Figure 9, during the initial stage of treatment (10 min), the proton density signal was primarily concentrated in the outer layers of the rice grains, while the signal in the central region was relatively weak. This indicates that at this stage, water was mainly distributed on the surface of the grains and had not yet fully penetrated into the interior. As the OH treatment time increased from 12 to 18 min, the signal intensity in the central region gradually increased, accompanied by a color transition from yellow-green to deep red, suggesting progressively improved moisture penetration and a more uniform internal water distribution. These observations indicate that OH treatment accelerated the hydration process within the rice grains. The more uniform moisture distribution is likely associated with the rapid volumetric heating characteristics of OH treatment, which accelerate water absorption and starch hydration. Previous studies have shown that starch gelatinization is accompanied by granule swelling and progressive disruption of the ordered starch structure, facilitating water diffusion within the starch matrix [36]. The present MRI results are consistent with this hydration behavior, although the underlying structural changes were not directly evaluated by MRI.

When the treatment time was extended to 20 min, the internal relaxation signals of the rice grains further intensified, and their overall distribution pattern became closer to that of the control group (CK-25), indicating that OH treatment had achieved a high degree of uniform hydration within the rice grains. Combined with the previous LF-NMR results (Figure 8A), it can be inferred that during the OH treatment, rapid volumetric heating and electric field effects jointly promoted the loosening of the starch structure and water migration, reducing the degree of water binding to macromolecules, thereby improving the mobility and diffusion capacity of water molecules. As water continued to penetrate into the interior of the rice grains, the processes of starch gelatinization and internal structural reorganization were further accelerated, ultimately enabling OH treatment to achieve a cooking effect close to that of traditional steaming in a shorter time.

#### 3.3.4. Scanning Electron Microscope (SEM) Analysis

**Figure 10:**
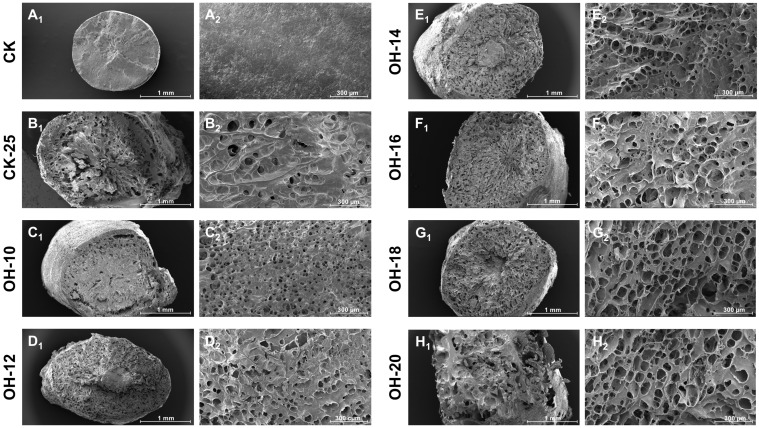
Scanning electron microscope images of indica rice with different treatment. (**A_1_**–**H_1_**) and (**A_2_**–**H_2_**) represent images observed at 200× and 1000× magnification, respectively.

Scanning electron microscope (SEM) images clearly reveal the dynamic evolution of the microstructure within indica rice during the OH treatment process (Figure 10). The cross-section of the untreated group exhibited an overall dense and continuous structure, with starch granules arranged in a regular and compact pattern and a relatively smooth surface; no obvious pores were observed. This indicates that, in its natural state, the interior of the rice grain retains an intact starch–protein composite structure, which strongly impedes water migration. After 10 min of OH treatment, localized cracks and slight structural loosening began to appear at the edges of the rice grain cross-section, indicating that rapid internal heating had already caused initial damage to the original dense structure. As the treatment time was further extended to 18 min, the number of pores within the cross-section increased significantly, gradually forming a honeycomb-like porous network structure, accompanied by localized fragmentation and collapse. This indicates that significant structural reorganization had occurred within the rice grain. Notably, according to the rapid heating profile (Figure 7), OH treatment quickly reached the gelatinization temperature range, indicating that thermal effects driven by volumetric heating and moisture vaporization are the primary contributors to early structural expansion and pore initiation. The formation of this porous structure is likely closely related to changes in internal pressure caused by rapid volumetric heating during the OH treatment process. During OH treatment, the moisture inside the rice grains heats up rapidly within a short period of time and undergoes localized vaporization. When the rate of internal heat generation exceeds the rate of moisture diffusion outward, the local vapor pressure gradually increases, causing the internal structure to expand and form a porous structure [40]. Concurrently, the non-thermal perforation effect generated by the pulsed electric field may trigger localized electrical breakdown at structural weak points, providing micron-scale pathways for early water penetration [41,42].

It is worth noting that although both the OH-18 and CK-25 groups exhibited significant starch granule swelling and structural loosening—indicating that both had reached a high degree of gelatinization—their microstructures still showed distinct differences. Compared to the CK-25 group, the OH-18 group displayed more pronounced porosity and localized structural fractures, suggesting that OH treatment formed a more open internal network structure. The pore network observed by SEM effectively reduces the resistance to water migration into the interior of the rice grains, thereby promoting more uniform internal hydration. This finding is consistent with the enhanced signal in the central region of the rice grains observed by MRI and the increased water mobility observed by LF-NMR. Furthermore, the looser starch–protein matrix facilitates further water absorption, swelling, and gelatinization of the starch granules, thereby accelerating the process of internal structural reorganization. Therefore, the porous structure formed by OH treatment is not only the result of microstructural disruption but also serves as a crucial structural foundation for promoting rapid cooking, water migration, and improved starch digestibility.

Furthermore, the formation of this porous structure may significantly influence the digestive characteristics and textural properties of cooked rice. On the one hand, the pore structure increases the contact area between water and digestive enzymes and the starch matrix, thereby enhancing starch accessibility and facilitating the digestive process; on the other hand, moderate structural loosening also helps improve the softness and masticatory properties of cooked rice [43]. However, for indica rice, which has a high amylose content and a relatively dense tissue structure, the specific contribution of electric field effects to structural remodeling requires further in-depth study.

#### 3.3.5. Analysis of Thermal Properties

Based on the thermogravimetric analysis results shown in Figure 8C,D, the thermal decomposition of rice in the temperature range of 30–600 °C can be divided into two main stages. The first stage (30–150 °C) primarily corresponds to the evaporation of free water and some low-molecular-weight volatile substances; the second stage (150–600 °C) is mainly associated with the thermal degradation of macromolecular components such as starch and proteins [28]. As shown in Table S8, compared with the untreated control (CK), the total weight loss rates of the CK-25 group and most OH-treated groups increased significantly. Specifically, the weight loss rate of the CK-25 group increased from 64.98% to 70.01%, while that of the OH-18 group increased by 4.01% compared with the OH-10 group, indicating that OH treatment significantly altered the thermal stability of the internal structure of rice grains.

The increased weight loss observed after OH treatment may be associated with changes in starch hydration, gelatinization, and structural organization during processing. Previous studies have reported that thermal treatment can modify the molecular arrangement and crystallinity of starch, thereby affecting its thermal degradation behavior [39]. It is worth noting that the decomposition temperature of the OH-20 group was significantly higher than that of the CK group, suggesting that a new, relatively stable structure may have formed within the rice grains after prolonged OH treatment. This phenomenon may be related to the rearrangement of starch molecular chains following gelatinization and enhanced starch–protein interactions [44]. Therefore, OH treatment not only leads to the gradual destruction of the original structure but is also accompanied by a process of new molecular network reconstruction, thereby resulting in a certain degree of improved thermal stability of the system under high-temperature conditions.

#### 3.3.6. Fourier Transform Infrared Spectroscopy (FTIR)

In the FTIR spectrum, the absorption peaks in the wavenumber range of 3000–3600 cm^−1^ primarily reflect the stretching vibrations of the OH and N-H bonds. The wavenumber shifts in this spectral region are closely related to the number of intramolecular hydrogen bonds [45]. The absorption peaks at 3360 cm^−1^ and 2945 cm^−1^ correspond to the O-H stretching vibration and the C-H bending vibration, respectively, while the characteristic peak at 1643 cm^−1^ is primarily associated with the H-O-H bending vibration of water molecules [46]. As shown in Figure 8B, all samples exhibited similar trends and no new characteristic peaks appeared, indicating that no significant covalent bond formation or chemical structural changes occurred during the rice treatment process, confirming that OH treatment is a physical modification process.

The characteristic absorption peaks at 1047 cm^−1^ and 1022 cm^−1^ correspond to the crystalline region (branched starch double-helix structure) and the amorphous region, respectively. The R (1047/1022 cm^−1^) ratio reflects the degree of ordered structure in starch molecules; a higher ratio indicates a larger proportion of crystalline regions [47]. As shown in Table S9, the R value for the untreated group (CK) was 2.17, indicating that the short-range ordered structure of natural indica rice starch remains relatively intact. After conventional cooking, the R value of the CK-25 group decreased by 35.94%, suggesting that thermal treatment substantially reduced the ordered arrangement of starch molecules. This decrease may be associated with starch hydration and gelatinization during heating, which are accompanied by swelling of starch granules and weakening of the interactions responsible for maintaining ordered structures.

It is worth noting that after 10 min of OH treatment, the R value of indica rice starch showed no significant difference from that of the CK-25 group, indicating that OH treatment can rapidly weaken the short-range ordered structure of starch within a short period of time. As the treatment time was further extended, the R value continued to decrease from 1.71 to 1.49, suggesting that OH treatment further promoted the transition of the ordered structure of starch molecules to a disordered state. This may be attributed to the rapid and uniform internal heating during OH treatment, which accelerates the motion of starch molecular chains, gradually disrupting the hydrogen bonds and weak interactions that maintain the stability of the double-helix structure. Consequently, this promotes the loosening of the crystalline structure and an increase in gelatinization degree [42]. Similar phenomena have also been observed in starch systems subjected to thermal processing and electric field-assisted treatment. Previous studies have shown that as the gelatinization degree increases, the 1047/1022 cm^−1^ ratio typically continues to decrease, reflecting the gradual disruption of local ordered structures [48,49,50]. Therefore, this study demonstrates that OH treatment not only affects the surface structure of starch granules but also further promotes the overall restructuring of the starch structure within the rice grains.

#### 3.3.7. X-Ray Diffraction (XRD)

**Figure 11:**
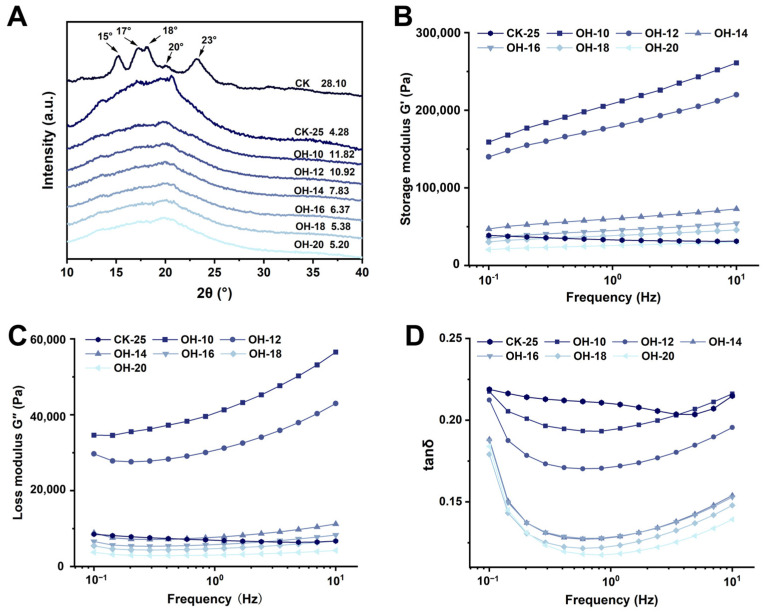
X-ray diffraction and rheological properties of indica rice subjected to different processing treatments. X-ray diffraction graph (**A**), indica rice G′ and G″ and tanδ (**B**–**D**). The control group is untreated and quickly cooked for 25 min.

XRD analysis provides direct evidence of changes in the long-range order of the starch in indica rice. As shown in Figure 11A, untreated indica rice exhibits a typical Type A XRD pattern, with distinct crystalline peaks observed at 2θ = 15°, 17°, 18°, and 23°. This is consistent with the crystallographic patterns of cereal starch reported in previous studies [26,51], with a crystallinity of 28.10. After cooking in a conventional rice cooker, the characteristic Type A peaks largely disappeared, and the crystallinity decreased significantly to 4.28%. This indicates that under the combined effects of high temperature and sufficient hydration, the starch granules underwent significant swelling, the original crystalline layer structure gradually disintegrated, and the long-range ordered arrangement was substantially weakened.

Compared with the conventional cooking group, OH-treated samples exhibited different diffraction characteristics. Weak diffraction peaks appeared near 2θ = 13° and 20°, indicating a transition toward a V-type crystalline structure. This phenomenon suggests that, during OH treatment, partial molecular rearrangement may occur after starch gelatinization, potentially involving the formation of amylose–lipid complexes as reported in thermally processed starch systems [52]. However, further molecular-level characterization would be required to confirm the specific composition and formation mechanism of these crystalline structures. This structural transformation is a common physical phenomenon in conventional thermal processing, further confirming that treatment under PEF conditions constitutes a physical denaturation process centered on thermal effects.

Combined with the FTIR results discussed earlier, it can be further observed that both the short-range ordered structure and the long-range crystalline structure of starch exhibit a synchronous decline during the OH treatment. The continuous decrease in the R-value in the FTIR spectrum indicates that the double-helix segments responsible for maintaining the ordered structure of starch are being progressively disrupted, causing the short-range arrangement to transition from ordered to disordered. Since the crystalline regions of starch essentially depend on the regular stacking of double-helix structures, the weakening of short-range order further undermines the stability of the crystalline layers, thereby leading to a decrease in long-range crystallinity as observed in XRD [53]. In other words, FTIR reflects the loosening of local molecular conformations, while XRD reflects the collapse of the crystal-level structure; together, they indicate that OH treatment triggers a top-down structural disintegration process, progressing from short-range to long-range.

At the same time, V-shaped diffraction peaks can still be observed in the OH-treated samples, indicating that while the structure is being disrupted, some chain segments have rearranged to form new composite crystals. This coexistence of “disruption and reorganization” also explains why the samples after OH treatment did not completely lose their ordered structure but instead transformed into a different crystalline morphology better suited for stable existence after cooking.

#### 3.3.8. Rheology Properties

Dynamic rheological results indicate that the storage modulus (G′) was consistently higher than the loss modulus (G″) in all samples, and tanδ was less than 1 (Figure 11B–D), suggesting that a gel network structure dominated by elastic behavior formed in the indica rice system after cooking. This phenomenon indicates that a continuous three-dimensional structure has formed within the system, in which the elastic energy storage capacity exceeds the viscous energy dissipation capacity. Previous studies have indicated that the extensive exudation of amylose during the cooking process and its subsequent intermolecular entanglement are crucial for the formation of an elastic gel network, a phenomenon that is particularly pronounced in systems with high amylose content [54,55]. During heating and hydration, starch granules gradually swell and gelatinize, releasing a portion of the amylose from within the granules. This amylose then forms a continuous network through hydrogen bonding, molecular entanglement, and interchain association, thereby imparting high elastic properties to the system.

To further compare the rheological differences among treatments, the values of G′, G″, and tanδ at 0.84 Hz (approximately 1 Hz) were selected from the frequency sweep curves for statistical analysis (Table S10). Significant differences were observed among different treatment groups, confirming that OH treatment duration significantly affected the viscoelastic properties of gelatinized rice systems.

Compared to steaming in a traditional rice cooker, OH-treated samples exhibited relatively higher G′ values, indicating that OH treatment is more conducive to the formation of a stable gel network structure. This may be related to the rapid and uniform volumetric heating characteristics during the OH treatment process. OH promotes the overall gelatinization of starch granules within a short period and enhances the migration and rearrangement capabilities of molecular chains, thereby increasing the degree of effective network connectivity. At the same time, moderate gelatinization helps to partially preserve the integrity of the starch molecular chains and promotes the formation of more effective physical cross-linking points within the system, thereby enhancing the elastic response. As shown in Figure 7, a faster heating rate shortens the residence time of the system in the intermediate phase, allowing the sample to rapidly enter the gelatinization temperature range. This facilitates the establishment of a more continuous and dense viscoelastic network structure at an early stage, providing a thermodynamic foundation for subsequent structural stability. Furthermore, the lower G″ values observed in the OH samples in this study indicate reduced viscous dissipation within the system, further suggesting that its internal network structure is more stable and continuous. This trend is consistent with the findings of Liu [56].

**Figure 12:**
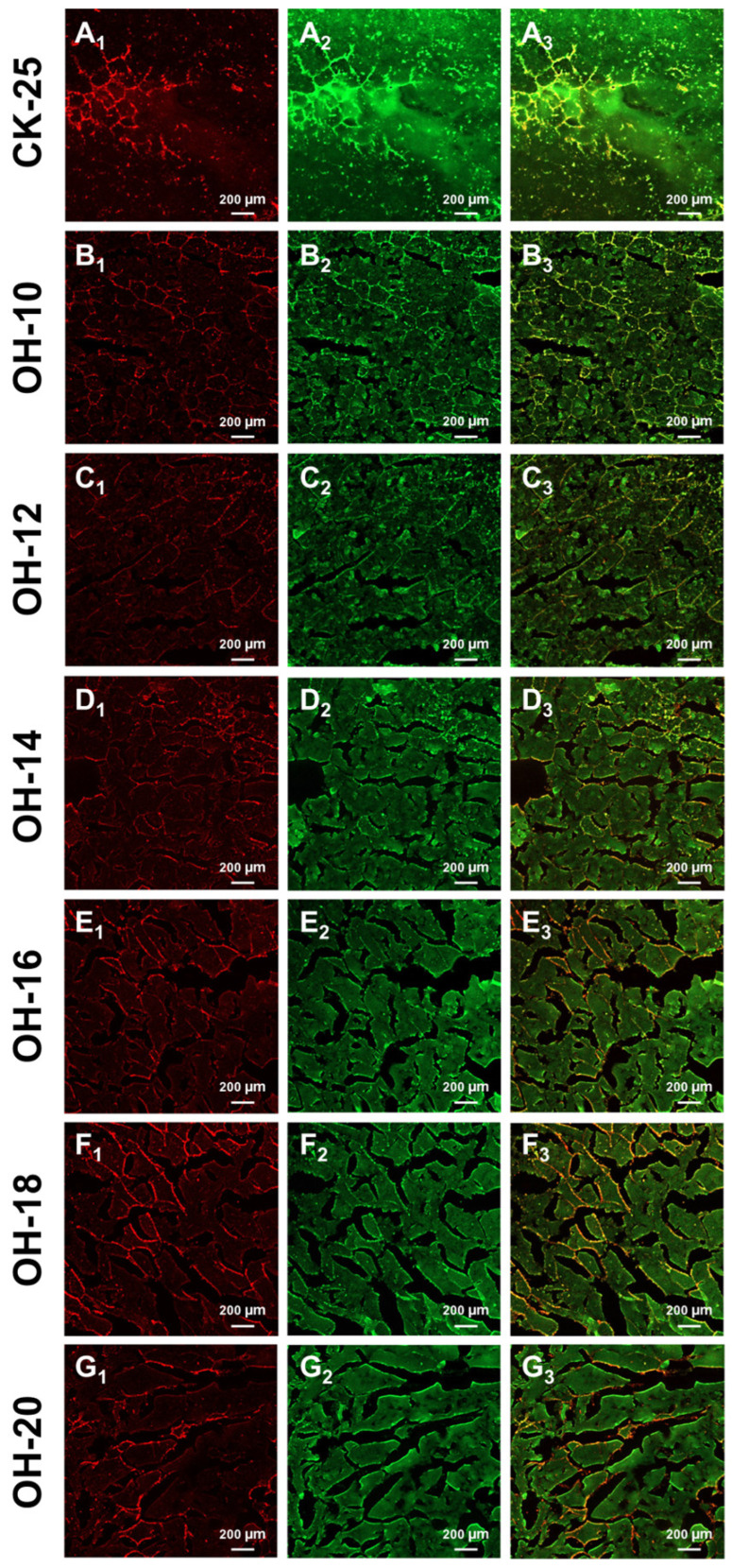
Confocal laser scanning microscope images of indica rice under different treatments. (**A_1_**–**G_1_**) and (**A_2_**–**G_2_**) represented the images observed at excitation wavelengths of 543 nm and 488 nm, respectively; (**A_3_**–**G_3_**) represented the images observed under multichannel superposition mode.

The formation of this elastic network may also be related to enhanced starch–protein interactions. CLSM results show more pronounced colocalization between starch and protein phases after OH treatment (Figure 12), suggesting that proteins may participate in gel network formation during the gelatinization process. Many researchers believe that starch–protein composite structures can increase the crosslinking density of the system and further enhance the stability and viscoelastic properties of the gel network [57,58]. Therefore, the elastic-dominated behavior observed in the OH-treated samples may be associated with starch chain reorganization together with increased spatial association between starch and protein phases. However, the molecular basis of these interactions requires further investigation.

It is worth noting that under constant frequency conditions, both G′ and G″ exhibit a gradual downward trend as the OH treatment time is further extended, which is consistent with the findings of [59]. This phenomenon suggests that while OH treatment promotes network formation, excessive treatment may lead to a decline in structural integrity. On the one hand, prolonged heating further weakens the residual crystalline structure; on the other hand, some starch molecular chains may degrade, reducing the effective chain length and thereby weakening the network’s supporting capacity [41].

#### 3.3.9. Confocal Laser Scanning Microscopy (CLSM)

Studies have shown that in confocal laser scanning microscopy (CLSM) images of the rice starch–protein complex, starch exhibits green fluorescence when it strongly binds to FITC, while proteins bind more readily to Rhodamine B, exhibiting red fluorescence. The region where green and red fluorescence overlap appears yellow [60]. CLSM images of CK-25 (Figure 12) reveal that, in the microstructure of fully matured rice grains, starch granules are tightly enveloped by a protein network. The images are predominantly green, indicating that starch is the major component. Simultaneously, partial colocalization of green and red fluorescence (yellow regions) is observable, suggesting an interaction between starch and proteins.

As the duration of OH treatment increased, the microstructure of the samples gradually transformed from discrete starch granules embedded in a protein matrix to a more uniform and continuous network structure. Particularly in the OH-18 group, the yellow overlapping regions further expanded, indicating an increased degree of spatial co-localization between the starch and protein phases. This suggests more thorough interfacial contact within the system, rather than a distinct phase-separated state. As starch granules continue to absorb water and swell, some amylose leaks out from within the granules, while the loosening of protein structures induced by the heating process may expose more binding sites capable of participating in non-covalent interactions, thereby promoting the formation of a tighter composite structure between the starch chains and the protein matrix [61].

The formation of this composite structure has a significant impact on the viscoelastic behavior of the system. A more uniform and continuous starch–protein network increases the number of effective connection points within the system, thereby enhancing the network’s ability to store elastic energy, which is consistent with the phenomena observed in the rheological results. Furthermore, the continuous structure formed after OH treatment may also reduce viscous energy dissipation within the system, causing the gel system to exhibit more pronounced elastic-dominated characteristics. At the same time, the disruption of native starch granules and the leaching of amylose further promote internal structural reorganization, leading to a more stable spatial distribution between the starch phase and the protein phase.

## 4. Limitations and Future Perspectives

Although this study provides comprehensive insights into the application of OH for rapid cooking of indica rice, several limitations should be acknowledged. First, only one indica rice cultivar was investigated in this study. Rice varieties with different amylose contents, protein compositions, grain structures, and physicochemical characteristics may exhibit distinct responses to OH treatment. Therefore, further investigations involving multiple rice cultivars with diverse starch properties are required to evaluate the general applicability of OH cooking and establish processing strategies suitable for different grain types.

Second, this study mainly characterized the structural evolution and quality changes of rice during OH treatment through multiscale approaches, including LF-NMR, MRI, SEM, FTIR, XRD, rheological analysis, and digestion evaluation. However, the molecular mechanisms underlying these structural transformations remain to be further clarified. Although the observed changes in starch ordering, water migration, and starch–protein spatial distribution provide evidence for OH-induced structural reconstruction, direct molecular-level evidence, such as starch molecular weight distribution, protein composition analysis, amino acid profiling, and proteomic characterization, was not obtained. In particular, the increased spatial association between starch and protein phases observed in this study does not directly demonstrate enhanced molecular interactions between starch and proteins. Further investigations using protein-specific analytical approaches, such as protein extraction, differential scanning calorimetry (DSC), Raman spectroscopy, or other protein-sensitive spectroscopic techniques, are required to verify the underlying molecular interactions. Future studies integrating omics approaches and molecular analyses are needed to further elucidate the specific pathways involved in OH-driven quality formation.

Third, although the electronic tongue and electronic nose analyses demonstrated differences in taste and volatile profiles between OH-treated and conventionally cooked rice, these techniques provide overall sensory response patterns rather than direct identification of specific flavor compounds or nutritional components. Therefore, future research should combine targeted metabolomic analysis, volatile compound identification, and free amino acid determination to further explain the contribution of OH treatment to flavor development.

Fourth, although OH has been widely recognized as an efficient volumetric heating technology, its electrical and thermal performance is strongly influenced by equipment configuration, electrode geometry, electrical properties of food materials, operating parameters, heating characteristics, and processing scale. The present study mainly focused on optimizing pulsed OH treatment conditions and evaluating their effects on cooking efficiency and quality characteristics of indica rice. However, several detailed electrical and thermal parameters, including real-time electrical conductivity changes during treatment, spatial temperature distribution and heating uniformity within the treatment chamber, current density profiles, and a complete electrical–thermal energy balance, were not evaluated due to limitations in the available monitoring equipment. Although the applied voltage, maximum current, and theoretical electrical energy input were estimated based on the operating parameters, direct measurements of electrical energy consumption, heat losses, and system conversion efficiency were not conducted. Future studies should incorporate advanced in situ electrical and thermal monitoring systems to characterize the dynamic behavior of OH processes and quantitatively evaluate heating uniformity, energy performance, and scale-up feasibility for rice cooking applications.

Fifth, although the sensory evaluation results provided valuable information regarding the overall quality perception of OH-treated rice, several limitations should be acknowledged. The sensory panel in this study consisted of 10 trained food science students, which was suitable for comparative evaluation of sensory attributes among different cooking treatments but may not fully represent the preferences of a broader consumer population. Moreover, although sample randomization, three-digit coding, blinded evaluation, and randomized serving order were implemented to minimize potential sensory bias, repeated evaluations using larger and more diverse consumer panels were not conducted. Future studies should incorporate expanded sensory panels with participants of different demographic backgrounds, together with consumer acceptance tests and preference analysis, to further validate the practical applicability and market acceptance of OH-cooked rice.

Finally, although the present study demonstrates that OH can significantly shorten cooking time while maintaining desirable quality characteristics, the relative contributions of thermal effects and electric field effects during OH processing remain difficult to completely separate. Since rapid volumetric heating is considered the dominant factor in OH cooking, further studies using controlled heating systems or comparative electric field treatments are necessary to clarify the specific role of electrical effects in starch gelatinization, moisture migration, and microstructural modification.

Overall, future research should focus on expanding the range of rice varieties, integrating molecular-level analytical techniques, and optimizing industrial-scale OH systems to promote the practical application of this technology in cereal processing.

## 5. Conclusions

In this study, the optimal OH processing conditions for indica rice were determined as 18 min treatment time, 9.4 μs pulse width, 0.52 kV/cm electric field strength, and 265 Hz pulse frequency. Under these conditions, compared with conventional electric rice cooking, the cooking time was shortened by approximately 28%, while achieving improved overall eating quality. Multiscale structural analyses indicated that OH processing was associated with accelerated starch gelatinization and structural reconstruction, accompanied by the disruption of starch crystalline regions, weakening of short-range and long-range ordered structures, enhanced moisture migration, and the formation of a more integrated starch–protein network. SEM and CLSM observations further revealed the development of a porous and continuous internal structure, which contributed to improved hydration uniformity and viscoelastic properties. In addition, FTIR and XRD analyses confirmed the transition of starch from an ordered crystalline structure to a more disordered gelatinized state, accompanied by the formation of V-type starch–lipid complexes.

Overall, this work reveals the multiscale structural changes associated with OH-cooked rice cooking and demonstrates its potential as an efficient physical processing strategy for improving the quality of cereal foods.

## Figures and Tables

**Table 1:** Design of single-factor experiments for OH parameter optimization.

Investigated Factor	Varied Levels	Fixed Parameters
Pulse Width	6, 7, 8, 9, 10 μs	Electric Field Strength: 0.35 kV/cm Treatment Time: 18 min Pulse Frequency: 300 Hz
Electric Field Strength	0.35, 0.40, 0.45, 0.50, 0.55 kV/cm	Treatment Time: 18 min Pulse Width: 9 μs Pulse Frequency: 300 Hz
Pulse Frequency	220, 240, 260, 280, 300 Hz	Electric Field Strength: 0.50 kV/cm Treatment Time: 18 min Pulse Width: 9 μs
Treatment Time	15, 16, 17, 18, 19 min	Electric Field Strength: 0.35 kV/cm Pulse Width: 9 μs Pulse Frequency: 300 Hz

**Table 2:** Variance analysis and significance test.

Source	Sum of Squares	df	Mean Square	F-Value	*p*-Value	
Model	246.33	14	17.59	21.37	<0.0001	significant
A Treatment time	19.94	1	19.94	24.23	0.0002	**
B Pulse width	13.59	1	13.59	16.51	0.0012	**
C Electric field strength	19.08	1	19.08	23.17	0.0003	**
D Pulse frequency	3.49	1	3.49	4.24	0.0586	
AB	0.0030	1	0.0030	0.0037	0.9525	
AC	0.3025	1	0.3025	0.3675	0.5541	
AD	1.90	1	1.90	2.31	0.1505	
BC	6.94	1	6.94	8.43	0.0115	*
BD	3.86	1	3.86	4.69	0.0481	*
CD	0.8649	1	0.8649	1.05	0.3227	
A^2^	81.81	1	81.81	99.38	<0.0001	**
B^2^	52.92	1	52.92	64.29	<0.0001	**
C^2^	66.17	1	66.17	80.38	<0.0001	**
D^2^	73.94	1	73.94	89.83	<0.0001	**
Residual	11.52	14	0.8232			
Lack of Fit	8.74	10	0.8742	1.26	0.4456	not significant
Pure Error	2.78	4	0.6956			
Cor Total	257.85	28				

Note: * Indicates significant difference (*p* < 0.05); ** Indicates highly significant difference (*p* < 0.01).

## Data Availability

The original contributions presented in this study are included in the article/Supplementary Materials. Further inquiries can be directed to the corresponding authors.

## Supplementary Materials

The following supporting information can be downloaded at: https://www.mdpi.com/article/10.3390/foods15152742/s1. Table S1. Electrical parameters and estimated energy input under different electric field strengths during OH treatment; Table S2. Indica rice response surface factor and level; Table S3. Sensory scoring criteria for rice; Table S4. Effect of OH treatment on color difference in indica rice; Table S5. The effect of OH treatment on the texture properties of indica rice; Table S6. Cooking quality and textural characteristics of indica rice under optimal OH Process; Table S7. Relaxation time of different treatments of indica; Table S8. Thermodynamic data of TGA curves of indica rice before and after OH treatment; Table S9. The infrared absorption ratio of rice under different treatment conditions was R (1047/1022 cm^−1^); Table S10. Rheological parameters of gelatinized rice paste at 0.84 Hz.
