# Supplementary material for: Application of Pulsed Ohmic Heating for Rapid Cooking of Indica Rice: Quality Enhancement and Mechanistic Insights
Source: Foods. 2026 Aug 4;15(15):2742. doi: 10.3390/foods15152742 (PMC13463885; doi:10.3390/foods15152742)
Supplement: Supplementary file 1 [file foods-15-02742-s001.zip › foods-4421913-supplementary.pdf]

Supplementary Materials

Table S1. Electrical parameters and estimated energy input under different electric field strengths during OH treatment

**Electric field strength (kV/cm)**	**Applied voltage (kV)**	**Maximum current (A)**	**Pulse width (μs)**	**Frequency (Hz)**	**Instantaneous power (kW)**	**Average power (W)**	**Treatment time (min)**	**Estimated energy input (kJ)**	**Energy input (kWh)**
0.35	1.05	16.00	9	300	16.80	45.36	18	48.99	0.0136
0.40	1.20	20.80	9	300	24.96	67.39	18	72.78	0.0202
0.45	1.35	23.20	9	300	31.32	84.56	18	91.32	0.0254
0.50	1.50	26.40	9	300	39.60	106.92	18	115.47	0.0321
**0.52**	**1.56**	**27.30**	**9.4**	**265**	**42.59**	**106.06**	**18**	**114.54**	**0.0318**
0.55	1.65	28.80	9	300	47.52	128.30	18	138.56	0.0385

Note: Bold values represent the optimized OH treatment condition. The estimated electrical energy input was calculated based on the peak current using the following equation: $Q = V \times I_{\max} \times PW \times f \times t$.

Table S2. Indica rice response surface factor and level

Level	Factors			
	A<br>Treatment time<br>（min）	B<br>Pulse width<br>（μs）	C<br>Electric field strength<br>（kV/cm）	D<br>Pulse frequency<br>（Hz）
-1	17	8	0.45	260
0	18	9	0.50	280
1	19	10	0.55	300

Table S3. Sensory scoring criteria for rice

Primary attribute	Secondary attribute	Sensory description	Score
**Aroma**	Authenticity and intensity	Characteristic rice aroma with strong and pleasant fragrance	18–20
		Characteristic rice aroma with moderate fragrance	15–17
		Characteristic rice aroma but weak intensity	12–14
		No obvious aroma but without off-flavor	7–12
		Presence of undesirable odor	0–6
**Appearance**	Color	Bright white and attractive appearance	6–7
		Normal color appearance	4–5
		Yellowish or grayish appearance	0–3
	Gloss	High surface gloss	7–8
		Slight gloss	5–6
		Lack of gloss	0–4
	Grain integrity	Compact structure with intact rice grains	4–5
		Mostly intact grains with relatively compact structure	3
		Grain cracking or burst appearance	0–2
**Palatability**	Stickiness	Smooth texture with appropriate stickiness and no adhesion to teeth	8–10
		Slightly sticky texture with little adhesion	6–7
		Excessive stickiness causing tooth adhesion or insufficient stickiness	0–5
	Elasticity	Good chewiness and elasticity	8–10
		Moderate chewiness	6–7
		Loose, hard, or coarse texture	0–5
	Hardness	Appropriate softness and firmness	8–10
		Slightly hard or slightly soft	6–7
		Excessively hard or soft texture	0–5
**Taste**	Authenticity and persistence	Rich rice flavor with pleasant sweetness during chewing	22–25
		Mild rice flavor with slight sweetness	18–21
		No obvious rice flavor or sweetness but without off-flavor	16–17
		No pleasant flavor and/or presence of off-flavor	0–15
**Cold rice texture**	Cohesiveness, elasticity, and hardness	Loose structure, good cohesiveness and elasticity, moderate hardness	4–5
		Slight clumping, reduced elasticity, and slightly increased hardness	2–3
		Severe clumping, poor elasticity, and excessive hardness	0–1

Table S4. Effect of OH treatment on color difference in indica rice

Time	*L**	*a**	*b**	*W*
CK-25	70.56 ± 0.32^{b}	-1.84 ± 0.02^{d}	1.78 ± 0.10^{e}	75.17 ± 0.65^{b}
IROH -10	74.86 ± 0.43^{a}	-1.66 ± 0.02^{cd}	2.98 ± 0.14^{d}	74.92 ± 0.45^{a}
IROH -12	71.99 ± 0.72^{c}	-1.68 ± 0.05^{c}	4.12 ± 0.32^{c}	72.57 ± 0.76^{c}
IROH -14	69.82 ± 0.14^{d}	-1.41 ± 0.01^{a}	3.51 ± 0.06^{d}	69.80 ± 0.29^{d}
IROH -16	67.41 ± 0.24^{e}	-1.27 ± 0.02^{a}	7.74 ± 0.20^{a}	68.09 ± 0.31^{e}
IROH -18	67.92 ± 0.65^{e}	-1.53 ± 0.21^{b}	6.33 ± 0.31^{b}	68.99 ± 0.03^{e}
IROH -20	71.02 ± 0.67^{d}	-0.75 ± 0.12^{e}	6.10 ± 0.64^{bc}	70.06 ± 0.09^{e}

Note: Samples means with different letters in the same column were significantly different at $P < 0.05$.

Table S5. The effect of OH treatment on the texture properties of indica rice

Groups	Hardness (gf)	Springiness	Chewiness (gf)	Gumminess (gf)	Cohesiveness	Resilience
CK-25	508.85 ±7.35^{b}	0.65 ± 0.02^{a}	182.81 ± 6.31^{b}	279.23 ± 6.78^{b}	0.53 ± 0.028^{a}	0.33 ± 0.02^{a}
IROH-10	2042.06 ± 89.24^{a}	0.35 ± 0.03^{c}	588.38 ± 13.63^{a}	1273.15 ± 22.68^{a}	0.54 ± 0.010^{a}	0.34 ± 0.019^{a}
IROH -12	659.33 ± 17.88^{b}	0.37 ± 0.01^{bc}	133.27 ± 5.51^{c}	245.76 ± 3.08^{bc}	0.34 ± 0.015^{b}	0.30 ± 0.012^{ac}
IROH -14	508.59 ± 8.63^{b}	0.43 ± 0.02^{bc}	138.70 ± 5.05^{c}	168.77 ± 7.45^{e}	0.36 ± 0.020^{b}	0.23 ± 0.011^{c}
IROH -16	512.87 ± 13.86^{b}	0.44 ± 0.01^{b}	91.29 ± 2.48^{d}	176.63 ± 6.01^{de}	0.39 ± 0.025^{b}	0.25 ± 0.018^{bc}
IROH -18	343.86 ± 5.04^{c}	0.41 ± 0.02^{bc}	88.03 ± 3.70^{d}	167.12 ± 5.75^{e}	0.38 ± 0.015^{b}	0.23 ± 0.011^{c}
IROH -20	330.11 ± 12.74^{c}	0.58 ± 0.01^{a}	112.84 ± 5.55^{cd}	219.54 ± 8.35^{cd}	0.52 ± 0.010^{a}	0.31 ± 0.025^{ab}

Note: Samples means with different letters in the same column were significantly different at $P < 0.05$.

Table S6. Cooking quality and textural characteristics of indica rice under optimal OH Process

Groups	Water Absorption Rate (%)	Volume Expansion Rate (%)	Hardness (gf)	Springiness	Chewiness (gf)	Adhesiveness (gf)	Cohesiveness	Resilience
Control	57.28 ± 0.73^{b}	268.25 ± 8.20^{b}	504.90 ± 20.65^{a}	0.77 ± 0.02^{a}	167.4 ± 13.46^{a}	285.79 ± 3.79^{a}	0.59 ± 0.01^{a}	0.35 ± 0.01^{a}
Best-OH	62.72 ± 0.7^{a}	372.54 ± 9.52^{a}	429.53 ± 18.41^{b}	0.50 ± 0.04^{b}	89.93 ± 20.31^{b}	199.46 ± 2.72^{b}	0.40 ± 0.05^{b}	0.26 ± 0.03^{b}

Note: Samples means with different letters in the same column were significantly different at $P < 0.05$.

Table S7. Relaxation time of different treatments of indica

	Total peak area (A)	T21 (ms)	A21	T22 (ms)	A22
CK	1791.56 ± 1.39^{e}	0.69 ± 0.02^{e}	1782.85 ± 5.36^{a}	124.40 ± 17.96^{a}	24.71 ± 0.04^{f}
CK-25	6899.70 ± 20.95^{d}	0.89 ± 0.03^{e}	949.94 ± 60.93^{b}	30.03 ± 1.22^{f}	6019.16 ± 53.48^{e}
OH-10	7580.24 ± 180.70^{c}	1.69 ± 0.07^{cd}	848.36 ± 59.09^{c}	43.49 ± 0.04^{e}	6814.88 ± 103.81^{d}
OH-12	7488.79 ± 32.68^{c}	1.58 ± 0.11^{d}	759.95 ± 12.56^{d}	41.50 ± 0.11^{e}	6742.83 ± 69.51^{d}
OH-14	8195.90 ± 127.04^{b}	1.68 ± 0.12^{d}	679.32 ± 44.74^{f}	48.97 ± 1.98^{de}	7283.59 ± 210.08^{c}
OH-16	8156.10 ± 58.47^{b}	2.02 ± 0.08^{bc}	567.39 ± 19.70^{e}	53.10 ± 2.27^{cd}	7482.71 ± 52.98^{b}
OH-18	8146.88 ± 169.77^{ab}	2.07 ± 0.15^{b}	554.67 ± 28.08^{e}	59.19 ± 2.54^{bc}	7599.21 ± 91.12^{b}
OH-20	8388.11 ± 78.3^{a}	2.38 ± 0.33^{a}	518.67 ± 8.15^{e}	67.47 ± 0.1^{b}	7899.64 ± 41.15^{a}

Note: Samples means with different letters in the same column were significantly different at $P < 0.05$.

Table S8. Thermodynamic data of TGA curves of indica rice before and after OH treatment

	Initial temperature (℃)	Termination temperature (℃)	Loss rate (%)
CK	298.81±3.11[a]	329.27±2.13[b]	64.98±1.89[b]
CK-25	301.49±2.62[a]	332.13±1.56[ab]	70.01±0.74[a]
OH-10	298.27±2.45[a]	330.38±1.48[b]	67.57±1.02[ab]
OH-12	298.40±0.98[a]	331.32±2.65[ab]	69.81±0.67[a]
OH-14	298.46±1.67[a]	331.35±3.48[ab]	69.36±0.99[a]
OH-16	298.99±2.45[a]	331.64±2.85[ab]	68.05±1.32[ab]
OH-18	300.24±2.05[a]	333.83±0.85[ab]	71.58±2.45[a]
OH-20	300.40±1.77[a]	337.24±1.89[a]	68.52±1.64[ab]

Note: Samples means with different letters in the same column were significantly different at $P < 0.05$.

Table S9. The infrared absorption ratio of rice under different treatment conditions was R (1047/1022cm^{-1})

	R (1047/1022cm^{-1})
CK	$2.17±0.11^{a}$
CK-25	$1.39±0.31^{b}$
OH-10	$1.71±0.09^{b}$
OH-12	$1.68±0.18^{b}$
OH-14	$1.54±0.20^{b}$
OH-16	$1.50±0.09^{b}$
OH-18	$1.50±0.16^{b}$
OH-20	$1.49±0.03^{b}$

Note: Samples means with different letters in the same column were significantly different at $P < 0.05$.

Table S10. Rheological parameters of gelatinized rice paste at 0.84 Hz

Sample	G′ (Pa)	G″ (Pa)	tanδ
CK-25	33266 ± 1258^{c}	7009 ± 312^{c}	0.211 ± 0.009^{a}
OH-10	205000 ± 6252^{a}	39561 ± 1850^{a}	0.193 ± 0.005^{ab}
OH-12	176000 ± 5487^{a}	30045 ± 1429^{b}	0.171 ± 0.008^{b}
OH-14	59354 ± 2174^{b}	7571 ± 352^{c}	0.128 ± 0.004^{c}
OH-16	44402 ± 1757^{c}	5678 ± 286^{d}	0.128 ± 0.005^{c}
OH-18	37845 ± 1423^{c}	4618 ± 232^{d}	0.122 ± 0.001^{c}
OH-20	25347 ± 983^{d}	2979 ± 161^{e}	0.118 ± 0.013^{c}

Note: Different lowercase letters indicate that there are significant differences ($p < 0.05$) between samples for the same parameter.
